# Timely initiation of breastfeeding and associated factors among mothers in Motta town, East Gojjam zone, Amhara regional state, Ethiopia, 2015: a cross-sectional study

**DOI:** 10.1186/s12884-016-1108-4

**Published:** 2016-10-19

**Authors:** Tilahun Tewabe

**Affiliations:** Bahir Dar University, College of Medicine and Health science, Bahir Dar, Ethiopia

**Keywords:** Timely initiation of breastfeeding, Prevalence, Associated factors, Motta, Ethiopia

## Abstract

**Background:**

Timely initiation of breastfeeding within one hour after birth and exclusive breastfeeding is recommended for the first six months of infant life along with continuation of breastfeeding up to two years. Timely initiation of breastfeeding has the potential to prevent 22 % of neonatal deaths. The objective of this study was to assess timely initiation of breastfeeding and associated factors among mothers who have infants less than six months of age in Motta town, East Gojjam, Amhara Regional State, Ethiopia.

**Method:**

Community based quantitative cross-sectional study was conducted from April 7, 2015 to May 7, 2015. Simple random sampling technique was applied after taking all registered mothers who have infants less than 6 months old from local health extension workers of each kebele. A total of 423 mothers with infant less than six month old were included in this study. The data was collected from all four Kebeles using interviewer administered questionnaire. Descriptive and inferential statistics were used to present the data. Both bivariate and multivariate logistic regression analyses were used to identify factors associated with timely initiation breastfeeding.

**Result:**

Prevalence of timely initiation of breastfeeding was78.8 % [95 % CL: 74.88 %, 82.72 %]. Mothers who gave birth to their infant in a health institution [AOR = 3.486(1.253, 9.700)], birthed vaginally [AOR = 5.722(3.134, 11.246)] and didn’t give prelacteal food [AOR = 4.627(2.095, 10.220)] were more likely to initiate breastfeeding early than their counterparts.

**Conclusion:**

Prevalence of timely initiation of breastfeeding in the study area was 78.8 %. Place of delivery, mode of delivery and prelactal feeding were the independent predictors of timely initiation of breastfeeding. Recommendations to increase timely initiation of breastfeeding were: encouraging mothers to deliver their child in a health institution, minimizing caesarean delivery as much as possible and educating mothers and community as a whole to avoid traditional prelactal feeding practice.

## Background

It is estimated that every day, as many as 4,000 infants and young children die worldwide because they are not breastfed [1]. World Health Organization recommends initiation of breastfeeding within one hour after birth, exclusive breastfeeding for the first six months of infant life and continuation of breastfeeding up to two years [2]. Timely initiation of breastfeeding has the potential to prevent 22 % of neonatal deaths if all infants were breastfed within an hour after birth [3]*.*

Early breastfeeding provides numerous benefits to infants, women, and society. It creates a special bond between mother and infant, enhances dental development, reduces risk for allergies, aids in cognitive development, and decreases the risk for obesity in later life. It also helps the uterus return to pre-pregnancy size faster: reduces risk of breast, ovarian, and uterine cancers: decreases the risk for osteoporosis; enhances emotional health, and saves money [4, 5].

Many factors have found to affect timely initiation of breastfeeding such as; societal beliefs favoring to start with other feeding, lack of adequate support in health facilities and in the community, aggressive promotion of infant formula through medias, and lack of knowledge on the dangers artificial feeding among women, their partners, and families [6].

The Ethiopian Demographic and Health Survey estimated approximately 51.5 % of newborns were initiated to breastfeeding within one hour of birth [7]. Ethiopian HSDP IV planned to increase in the proportion of timely initiation of breastfeeding to 92 % by the end of 2015 [8].

Breastfeeding and good nutrition for children are recognized as essential for achieving the Millennium Development Goals (MDG), particularly the goals relating to child survival, such as reducing child mortality by 2/3 between 1990 and 2015 [9].

Therefore, this study was conducted in Motta town to assess early initiation of breastfeeding and associated factors among mothers of children aged less than 6 months.

## Methods

### Study setting and participants

Community based quantitative cross-sectional study was conducted from April 7 to May 7, 2015. The study was conducted in Motta town which is located in Amhara National Regional State, East Gojjam Zone, Ethiopia. It is bordered in all dimensions by Hulet Ejju Enesse woreda. It is 371 km away from Addis Ababa. Motta town was founded in 1754. The town has a total of 4 kebeles. The population of the town is 33,500. The town has a total of 17 governmental and nongovernmental health care institutions. There is a hospital, health center, five clinics, a pharmacy and nine drug stores.

The sample size was calculated using single population proportion formula by considering the following assumptions; P (proportion of timely initiated mothers) = 50 % to get maximum sample size to represent the community, 95 % level of confidence 95 %, level of significance = 5 %, margin of error (d) = 5 and 10 % non- response rate. Using simple random sampling technique 423 mothers participated in the study. To select study participants from each kebele first the sample size was propertionally allocated to size and then a lottery method was used.

#### Measurement

A structured interviewer administered questionnaire was used to collect data from participants or mothers of a child. The questionnaire was constructed by adopting from Ethiopia Demographic and Health Survey (EDHS) 2011 and from previous research done on similar topic and modified accordingly.

A one day infant diet recall method was used for assessing exclusive breastfeeding. Four diploma nurses were recruited as data collectors and two Bachelor of Science nurses were recruited as supervisors. All data collectors and supervisors were oriented and trained on how to interview and record the data one day before the survey.

### Operational definitions

#### Timely initiation of breastfeeding

If an infant within one hour of birth is put on mother’s breast to feed.

#### Pre-lacteal feeding

If an infant within the first three days of life was fed something other than breast milk.

#### Adequate knowledge about breast feeding

If a mother answered half or more questions correctly, from questions which were asked to measure breastfeeding knowledge, then adequate knowledge was determined.

### Statistical analysis

The collected data was checked manually for completeness and consistencies, and then it was coded and entered in EPI Info version 3.5.3 and exported to SPSS version 16 for analysis. Descriptive statistics was used to summarize the socio-demographic characteristics’ of the study participants and the prevalence of timely initiation of breast feeding. To identify factors associated with timely initiation breastfeeding practice, binary logistic regression analysis carried out at two levels, first bivariate logistic regression was performed to each independent variable with the outcome variable and those variables with a p value < 0.05 was included in the final model (multivariate analysis). Strength of association was measured using odds ratio, and 95 % confidence intervals. Statistical significance was declared at P value < 0.05.

Ethical clearance was obtained from Addis Ababa University, department of nursing and midwifery research committee. Each study participant was adequately informed about the objective of the study and anticipated benefit and risk of the study by their data collector. Verbal consent was obtained from study participants for protecting autonomy and ensuring confidentiality. Respondents were also told of their right not to respond to the questions if they did not want to respond or if they wished to terminate the interview at any time.

## Results

### Socio-demographic characteristics

Out of 423 eligible mothers, 405 agreed to participate in this study, which made a response rate of 95.7 %. Age of mothers ranges from 18 to 47 years, with median age of 27 year. Around one third 127 (31.4 %) of mothers were between 25 and 29 years. More than half 229 (56.5 %) of mothers were Orthodox Christian followers. With regard to educational status, 162(40.2 %) mothers received no education. The majority 323 (79.6 %) of study participants were unemployed mothers. From all, 328(80 %) mothers live in a nuclear family. The average household income of the respondents was 76.22 USD month (SD ± 62.98), and 198(48.9 %) respondents earn less than 50 USD per month (Table 1).

**Table 1:** Socio-demographic characteristics mothers (respondents) who have infants less than six months old, in Motta town, East Gojjam Zone, Ethiopia, 2015

Variable	Category(*n* = 405)	Frequency	Percent (%)
Age of mother(in years)	15–19	5	1.2
	20–24	106	26.2
	25–29	127	31.4
	30–34	94	23.2
	35 and above	73	18.0
Religion	Orthodox	229	56.5
	Muslim	158	39
	Others^a^	18	4.4
Ethnicity	Amhara	388	95.8
	Others^b^	17	4.2
Level of education of mother	No education	163	40.2
	Primary level (1–8 grade)	120	30.0
	High school and above	122	29.8
Occupational status of mother	Employed	82	20.3
	Unemployed	323	79.6
Current marital status	Married	347	85.7
	Unmarried^c^	58	14.3
Husband educational level	No education	79	22.4
	Primary level (1–8 grade)	131	37.1
	High school and above	143	40.5
Husband occupation	Employed	111	31.5
	Unemployed	294	68.5
Type of family	Nuclear family	328	80
	Extended family	77	20
Household income/month/	<50 USD^d^	198	48.9
	50–100 USD	107	26.4
	>100 USD	100	24.7

^a^Protestant and Catholic ^b^Oromo, Tigrie and Gurage ^c^single, widowed and separeted ^d^united states dollar

### Infant and maternal health service utilization characteristics

Almost half 211 (52.1 %) of mothers have 2–3 children. One hundred ten (27.2 %) infants were first in birth order. Majority 349 (86.2 %) of mothers received antenatal care (ANC) during period of pregnancy out of them only 206 (59.0 %) were counseled concerning to optimal breast feeding practice. With regarding to place of delivery, most 338 (83.5 %) mothers delivered in a health institution. From total respondents, around half 197 (48.6 %) of mothers received postnatal care (Table 2).

**Table 2:** Infant and maternal health service utilization characteristics of study participants in Motta town, East Gojjam zone, Ethiopia, 2015

Variable	Response (*n* = 423)	Frequency	Percent (%)
Number of children	One	111	27.4
	Two to three	211	52.1
	Four and above	83	20.5
Sex of child	Male	180	44.4
	Female	225	55.6
Age of child	0–1 month	55	13.6
	2–3 month	119	29.4
	4–5 month	231	57.0
Birth order	First	110	27.2
	Second	125	30.9
	Third and above	169	41.9
Birth interval	Up to three years	236	58.3
	Three years and above	169	41.7
ANC follow up	Yes	349	86.2
	No	56	13.8
Number of ANC follow up (*n* = 349)	Less than or equal to three	86	14.6
	Four times	263	75.4
Counseling related breast feeding during ANC(*n* = 349)	Yes	206	59.0
	No	143	41.0
Place of birth	Health facility	338	83.5
	Home	67	16.5
Mode of delivery	Vaginal /normal	367	90.6
	Caesarean section	38	9.4
PNC	Yes	197	48.6
	No	208	51.4
Counseling regarding to breastfeeding during PNC(*n* = 197)	Yes	186	94.4
	no	11	5.6

### Breastfeeding and related practices

From total, 320 (78.8 %) mothers initiated breast milk to infant immediately within one hour of birth. Most mothers 323 (79.8 %) fed colostrum/first milk to the newborn. Majority 323 (79.8 %) of mothers didn’t give prelactal food other than breast milk within three days of an infant life. Prevalence of timely initiation of breastfeeding in this study was 78.8 %. Among mothers who didn’t initiate breastfeeding timely to the newborn, the main reasons mentioned were; caesarean section 28 (32.9 %), baby was sick 12 (14.1 %), mother was sick 15 (17.6 %) and delayed milk secretion 30 (35.3 %) (Table 3).

**Table 3:** Breastfeeding related practices of mothers who have infants less than six months old in Motta town, East Gojjam zone, Ethiopia, 2015

Variables	Responses (*n* = 405)	Frequency	Percent (%)
Breastfeeding experience of current infant	Yes	405	100
	No	0	0.0
Timely initiation of breastfed	Immediately with in 1 h	319	79.0
	1 h to 1 day	78	19.3
	After one day	8	2
Reasons for not initiating within one hour	Caesarean section	28	32.9
	Baby was sick	12	14.1
	Mother was sick	15	17.6
	Delayed milk secretion	30	35.3
Colostrum feeding	Yes	323	79.8
	No	82	20.2
Prelacteal feeding	No	323	79.8
	Yes	82	20.2

### Knowledge and informational status of mothers regarding to breastfeeding

**Fig. 1:**
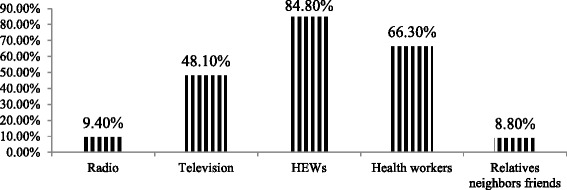
Sources of information for mothers about breastfeeding who have infant less than six months old in Motta town, East Gojjam zone, Ethiopia, 2015

Almost two thirds, 283 (69.9 %), of mothers know that breastmilk is initiation within one hour of birth of infant is recommended and 310 (76.5 %) mothers had adequate knowledge regarding to breastfeeding. Regarding to information about exclusive breastfeeding 362(89.4 %) mothers were informed about EBF from different sources (Fig. 1).

### Factors associated with timely initiation of breastfeeding

From total participants of this study, 79 % of mothers initiated breastfeeding to thier newborn within one hour of birth. The independet predictors for timely initiation of breastfeeding were; marital status, number of children, antenatal care, counseling about breastfeeding during antenatal care, place of birth, mode of delivery and husband support.

The final predictors for timely initiation of breastfeeding were counseling during ANC, place of birth and mode of delivery.

Breastfeeding counseling during pregnancy facilitates mothers to initiate breastfeeding immediately after birth of newborn. Mothers who were counseled regarding breastfeeding during ANC were almost 6 times more likely to initiate breastmilk immediately after birth of infant than mothers who were not counseled [AOR = 6.684(3.557, 12.58)].

Whereas mothers who gave birth in a health facility were almost three times more likely to initiate breastfeeding than those mothers who birthed their child at home [AOR = 2.831(1.134,7.066].

Mode of delivery was also significantly associated with timely initiation of breastfeeding. Mothers who experienced a normal vaginal dilevery were almost 4 times more likely to initiate breastfeeding immediately when compared to mothers who delivered by caesarean section [AOR = 3.722(1.484, 9.334)] (Table 4).

**Table 4:** Factors that affect timely initiation breastfeeding among mothers of infants age less than 6 months using bivariate and multivariate logistic regression analysis model, East Gojjam, Ethiopia, 2015

Variables	Timely initiation of breastfeeding					
		Yes (N & %)	No (N & %)	COR (95 % CL)	AOR(95 % CL)	p- value
Marital status	Married	280(80.7)	67(19.3)	2.036(1.107, 3.746)		
	Unmarried	38(67.2)	19(32.8)	1		
Number of children	1	90(81.1)	21(18.9)	1.955(1.006,3.797)		
	1–3	172(81.5)	39(18.5)	2.012(1.127,3.592)		
	4+	57(68.7)	26(31.5)	1		
ANC	Yes	282(80.8)	67(19.02)	2.161(1.171,3.994)		
	No	37(66.1)	19(33.9)	1		
Breastfeeding counseling during ANC	Yes	177(85.9)	75(14.1)	2.209(1.287, 3.791)		
	No	105(73.4)	29(26.6)	1	1	
Place of birth	Health facility	278(82.2)	60(17.8)	2.938(1.670, 5.171)	3.486(1.253,9.700)*	**0.007**
	Home	41(61.2)	26(38.8)	1	1	
Mode of delivery	Normal/vaginal	313(85.3)	54(14.7)	6.442(3.124, 12.36)	5.722(3.134,11.246)*	**0.0001**
	C/S	16(42.1)	22(57.9)	1	1	
Colostrum feeding	Yes	270(83.6)	53(16.4)	3.431(2.018,5.832)		
	No	49(59.8)	33(60.2)	1		
Prelactal feeding	No	276(85.7)	46(14.3)	5.714(3.351,9.745)	4.627(2.095, 10.220)*	**0.0001**
	Yes	42(51.2)	40(48.8)		**1**	

*N* number, *%* percent

1 = reference

## Discussion

In spite of what is known about timely initiation of breastfeeding; the practice is not satisfactory in the study area. Only 78.8 % [95 % CL: 74.88 %, 82.72 %] of mothers reported early initiation of breastfeeding, which is lower than Ethiopian HSDP IV target level; i.e. to increase the proportion of timely initiation breastfeeding mother from 51.5 to 92 % by the end of 2015 [8]*.*

This result is higher when compared to the 2011 Ethiopian DHS report 51.5 % [7] and from studies done in; Saudi Arabia 22 % [10], Raya Kobo Northwest Ethiopia 71.7 % [11], Goba district Southeast Ethiopia 52.4 % [12], Arba Minch zuria southern Ethiopia 57.6 % [13], India 36.4 % [14], 34 % among those who deliver their child through vagina in Harcourt teaching hospital [15], Nepal 66.4 % [16], in a study using 2011 EDHS 52 % [17], Tanzania 51 % [18], Timor Liste 46.1 % [19], in Lebanon 55.9 % [20], in hospital delivered infants India 36.4 % [21], in baby friendly hospitals in Turkey 35.2 % [22].

Such variations may due to methodological variations between studies, dissimilarities in infant and maternal socio-demographic characteristics and other differences in sociocultural, economical, health and health service utilization characteristics between respondents of the referenced areas and the study place.

Among mothers who didn’t initiate timely breastfeeding, the main reasons mentioned were; caesarean section (32.9 %), baby was sick (14.1 %), mother was sick (17.6 %) and delayed milk secretion (35.3 %).

Place of delivery were significantly associated with timely initiation of breastfeeding. Mothers who deliver their child in health institution were almost 3.5 times more likely to practice timely initiation of breastfeeding than others. This finding is consistent with studies done in; Goba district Ethiopia [12], Nepal [16] and Tanzania [18]. This may be due to large number of home deliveries in the country and the cultural or traditional activities in the community that promote prelactal feeding to the infant before starting breast milk.

Mode of delivery was also significantly associated with timely initiation of breastfeeding in the study area. Mothers who gave birth vaginally were almost six times more likely to initiate breastfeeding than mothers who deliver through caesaraan section. This result is in line with studies done in Saudi Arabia [10], a systematic review done of Medline LILACS Scopus and Web of Science electronic databases [23], Harcourt teaching hospital [15], a study using 2011 EDHS [17], Tanzania [18], Lebanon [20] and Turkey [22]. This may be due to the effects of anesthesia delaying the onset of lactation. Appropriate guidelines for caesarean deliveries are needed to minimize delays in initiation of breastfeeding. Prospective mothers and health workers should be informed about the negative association between pre labor caesarean delivery decisions, and breastfeeding and the implications for infant wellbeing.

Whereas mothers who did not give prelacteal feeds were almost 4.6 times more likely to initiate breastfeeding within one hour than mothers who introduce other fluids, which is similar with findings in;- Raya kobo Ethiopia [11], western Nepal [24], Saudi Arabia [10] and India [21]. The introduction of prelacteal feeds may decrease infants suckling activity which in turn can affect or decrease maternal milk production due to decreased breast stimulation. The use of prelacteal feeding appears to be a constriant in the promotion of EBF. Explanations this study provided were delayed milk secretion; due to breast problems, illness of mother and culture/ traditional practices such as the belief that giving liquid will clean the baby’s throat. This habit harms the newborn and exposes him/her to various morbidities and therefore; the practice needs to be discouraged.

A limitation of this study is that only the quantitative aspects of timely initiation of breastfeeding were assessed. Also results may under estimate the prevalence of timely initiation of breastfeeding in the study area due to recall bais.

## Conclusion

The prevalence of timely initiation of breastfeeding in the study area was 78.8 %, which was lower than the country recommended level. Among different socio-demographic, health service, maternal, and infant related factors studied; the place of delivery, mode of delivery and not giving prelactal feeding were the determinant factors for higher chance of timely initiation of breastfeeding.

Recommendations for improving EBF include; behavior change communication to avoid traditional activities, minimizing indications of caesarean delivery by health professionals, training of health professionals regarding to infant feeding practices, community based breastfeeding education and counseling to pregnant women and encouraging all mothers to give birth in health facilities.

## Abbreviations

AAU: Addis Ababa University
ANC: Antenatal care
AOR: Adjusted odds ratio
CL: Confidence level
EDHS: Ethiopian Demographic Health Survey
MDG: Millenium Development Goals
PNC: Postnatal care
SD: Standard deviation
USD: United States Dollar
WHO: World Health Organization
